# Supplementary material for: Seminoma and Embryonal Carcinoma Footprints Identified by Analysis of Integrated Genome-Wide Epigenetic and Expression Profiles of Germ Cell Cancer Cell Lines
Source: PLoS One. 2014 Jun 2;9(6):e98330. doi: 10.1371/journal.pone.0098330 (PMC4041891; doi:10.1371/journal.pone.0098330)
Supplement: File S1 — ZIP file containing DMRforPairs output for significant regions. Please start from the html files. (ZIP) [file pone.0098330.s008.zip › figures/36926.pdf]

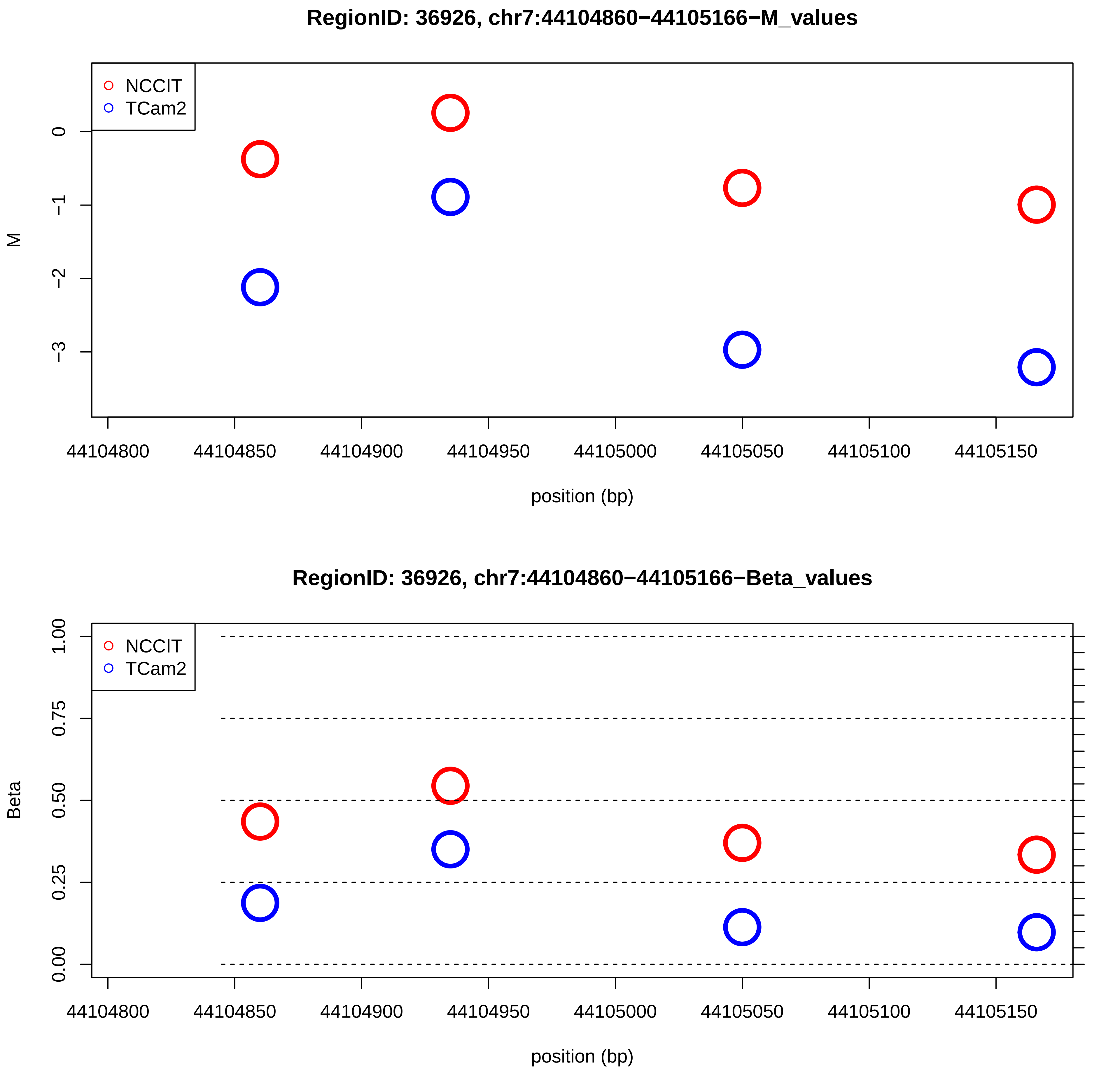
RegionID: 36926, chr7:44104860−44105166−M_values
NCCIT
TCam2
M
0
−1
−2
−3
44104800
44104850
44104900
44104950
44105000
44105050
44105100
44105150
position (bp)
RegionID: 36926, chr7:44104860−44105166−Beta_values
NCCIT
TCam2
Beta
0.00
0.25
0.50
0.75
1.00
44104800
44104850
44104900
44104950
44105000
44105050
44105100
44105150
position (bp)
